# Accurate machine learning-based germination detection, prediction and quality assessment of three grain crops

**DOI:** 10.1186/s13007-020-00699-x

**Published:** 2020-12-22

**Authors:** Nikita Genze, Richa Bharti, Michael Grieb, Sebastian J. Schultheiss, Dominik G. Grimm

**Affiliations:** 1 Technical University of Munich, TUM Campus Straubing for Biotechnology and Sustainability, Bioinformatics, Schulgasse 22, 94315 Straubing, Germany; 2 Weihenstephan-Triesdorf University of Applied Sciences, Petersgasse 18, 94315 Straubing, Germany; 3 Department of Informatics, Technical University of Munich, Boltzmannstr. 3, 85748 Garching, Germany; 4 Technology and Support Centre in the Centre of Excellence for Renewable Resources (TFZ), Schulgasse 20, 94315 Straubing, Germany; 5 Computomics GmbH, Eisenbahnstr. 1, 72072 Tübingen, Germany

**Keywords:** Seed germination, Germination prediction, Germination indices, Machine learning, Faster R-CNN

## Abstract

**Background:**

Assessment of seed germination is an essential task for seed researchers to measure the quality and performance of seeds. Usually, seed assessments are done manually, which is a cumbersome, time consuming and error-prone process. Classical image analyses methods are not well suited for large-scale germination experiments, because they often rely on manual adjustments of color-based thresholds. We here propose a machine learning approach using modern artificial neural networks with region proposals for accurate seed germination detection and high-throughput seed germination experiments.

**Results:**

We generated labeled imaging data of the germination process of more than 2400 seeds for three different crops, *Zea mays* (maize), *Secale cereale* (rye) and *Pennisetum glaucum* (pearl millet)*,* with a total of more than 23,000 images. Different state-of-the-art convolutional neural network (CNN) architectures with region proposals have been trained using transfer learning to automatically identify seeds within petri dishes and to predict whether the seeds germinated or not. Our proposed models achieved a high mean average precision (mAP) on a hold-out test data set of approximately 97.9%, 94.2% and 94.3% for *Zea mays*, *Secale cereale* and *Pennisetum glaucum* respectively. Further, various single-value germination indices, such as Mean Germination Time and Germination Uncertainty, can be computed more accurately with the predictions of our proposed model compared to manual countings.

**Conclusion:**

Our proposed machine learning-based method can help to speed up the assessment of seed germination experiments for different seed cultivars. It has lower error rates and a higher performance compared to conventional and manual methods, leading to more accurate germination indices and quality assessments of seeds.

## Background

Seeds are essential for human society as a food source and serve as starting material for crops. The yield of crops is not only highly dependent on environmental factors but also on the quality of the seed. Therefore, assessment of seed germination is an essential task for seed researchers to measure the performance of different seed lots in order to improve the efficiency of food chains [1]. In fact it has become imperative as the global crop production must be doubled in order to supply a rising population by 2050 [2].

Conventional seed testing measures, especially seed vigor tests, are not widely used due to cumbersome and time intensive protocols [3]. In addition, most seed tests developed by the International Seed Testing Association (ISTA) are evaluated manually using standardized procedures that differ for different crops [4].

In order to reduce the number of manual steps in seed testing, which is highly error-prone, many researchers have proposed methods to automate this process. Recently, modern image analysis techniques have been applied to detect seeds, because they can be easily automatized and provide unbiased and quantitative measurements with minimal errors [5–8]. However, most of the reported algorithms just use color-based thresholds on images and estimate factors to describe the seed, such as area, perimeter, length, width, roundness and color values [9]. GERMINATOR is a software that measures the area and the difference in position between points in time of images as an indicator for germination in *Arabidopsis thaliana* [10]. Importantly, for different seeds, several parameters require modifications and the system is most likely to fail with changes in illumination or partial occlusion of the seeds.

Similarly, Seed Vigor Imaging System (SVIS) processes RGB pixel values of digitally scanned images using a flatbed scanner to calculate the length of seeds [11]. On the one hand, when using a scanner instead of a camera, illumination settings are standardized, which improves performance. On the other hand, this method requires manual imaging of seeds and the researcher needs to be present throughout the germination experiment in order to assess the seeds.

Previously, assessment of multiple machine learning algorithms, Naive Bayes Classifier (NBC), k-Nearest Neighbour (k-NN), Decision Trees, Support Vector Machines (SVM) and Artificial Neural Networks (ANN) for comparing seed germination suggested higher performance and accuracy of ANN models [12]. Therefore the authors extracted 11 features using image processing, which is another manual step in germination tests.

In contrast, Deep Learning, especially Convolutional Neural Networks (CNNs), is a novel method to process images [13]. CNNs automatically extract and learn relevant features from raw images and have been applied to a large variety of image classification problems. One reason for their success is a lower dependency to different illuminations and obstructions, which leads to higher accuracy in computer vision tasks. CNNs have been already applied to automatically evaluate the germination rate of rice seeds [14]. However the images were only captured after the germination experiment was conducted, thus only the final germination percentage could be estimated with this approach.

The purpose of this study is to reduce the time-consuming and labor-intensive human visual inspections of seed germination experiments and to develop an improved germination prediction method that is (1) independent of custom color-based thresholds and thus can be applied to multiple seed cultivars and illumination settings and (2) can be used to better explore the dynamics of seed germination by estimating not only the final germination percentage but additional indices like rate and uniformity.

We present a machine learning-based method, using modern convolutional neural networks with region proposals, for an automated and high-throughput assessment of seed germination experiments for various species. For this purpose, we generated a labeled dataset of seeds and their germination process with more than 23,000 images for three different cultivars. We trained various deep learning models using transfer learning [15] in order to accurately detect seeds within an image, discriminate between their germination states, and finally to compute commonly used germination indices to measure the quality and dynamics of the seed lot. The proposed method could be implemented for improving scalability of automated seed germination assessments and to reduce errors in manual assessment of new seed lots.

## Methods

### Image acquisition

Seeds of either *Zea mays*, *Secale cereale* and *Pennisetum glaucum* were placed on petri dishes to capture a wide range of different germination states using digital imaging. Within the petri dishes the seeds were placed on a black cloth to ensure a high contrast between the emerging radicle and the background. The cloth was watered with tap water and petri dishes were covered with a lid to lower the effect of water evaporation and the resulting dry out of seeds. In a few cases the lid led to reflections, such as reflections of the camera setup (different examples are shown in Additional file 1: Figure S1).

**Fig. 1:**
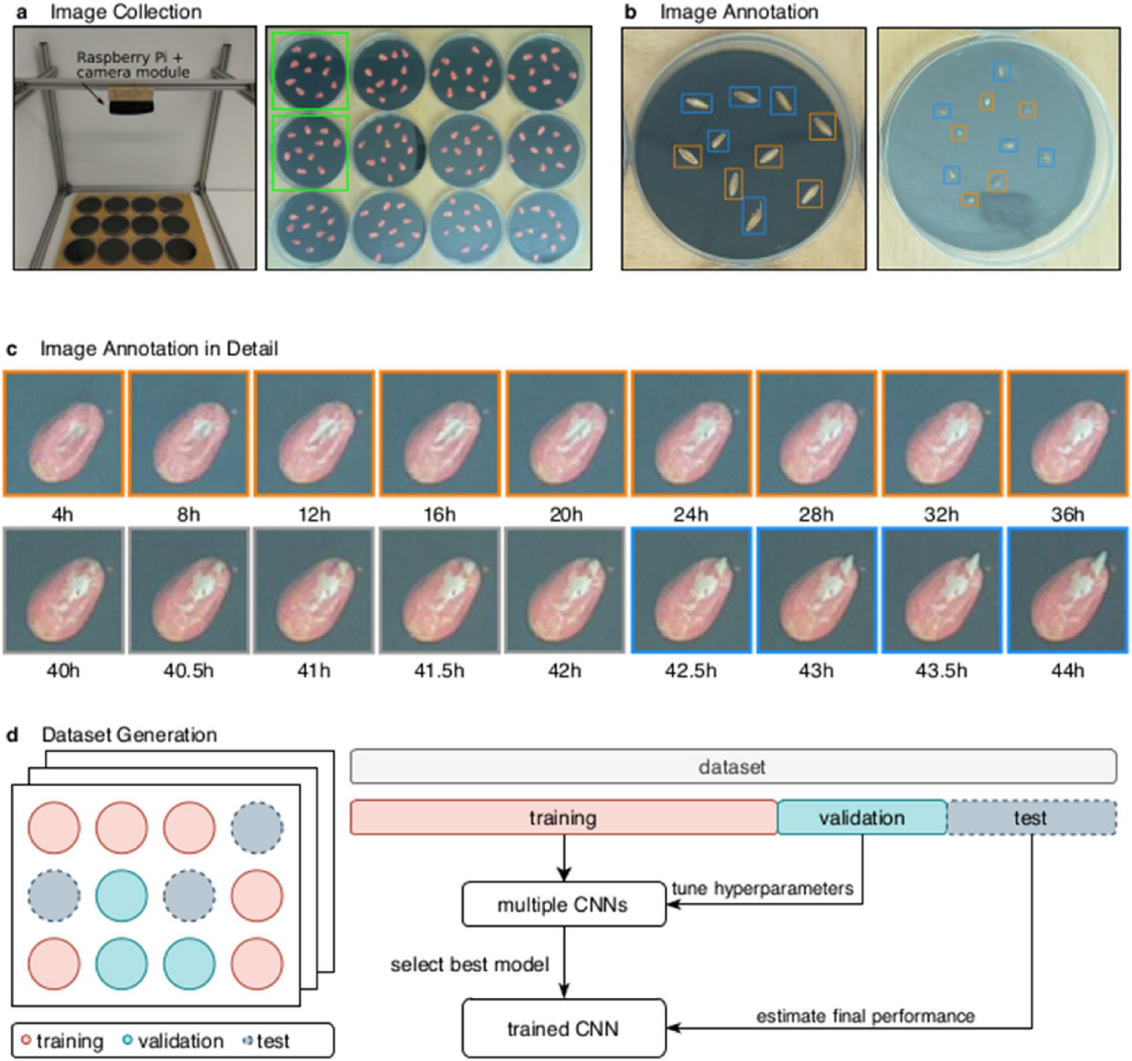
Illustration of image collection, annotation and dataset generation module. **a** Setup for capturing images of the germination process of seeds within petri dishes. Subsequently, images have been cropped to only contain one petri dish per image. **b** Example of annotated images, where seeds have been marked with a bounding box and a class label (non-germinated in orange, germinated in blue). **c** Longitudinal images of a custom seed for 48 h. Orange frames around the images indicate that the seed is not germinated, gray indicates a difficult to label transition phase and blue indicates that the seed is clearly germinated. **d** The dataset was randomly split into a training, validation and test set, stratified by petri dishes. This ensures that seeds of the same petri dish are either in the training, validation or test set. In addition, it also ensures that a petri dish at different time points only appears in one of the sets

All images were taken in an office at room temperature with the same artificial light source (4000 K Cool White fluorescent light bulb), which was turned on throughout the full capturing process (~ 48 h). For this purpose, twelve petri dishes were arranged in a 4 × 3 grid with an 8 megapixel camera module (Imaging Platform: CropScore Large-Scale Phenotyping System by Computomics GmbH, incorporating Raspberry Pi Camera Module v2.1) above the grid to capture the germination process, as illustrated in Fig. 1a. Seed Germination was captured over a time span of approx. 2 days with a resolution of two images per hour (every 30 min).

### Data preprocessing

Images were cropped into 624 × 624 pixel patches containing only one petri dish, as indicated in Fig. 1a. The open source software CVAT (https://github.com/opencv/cvat) was used to draw bounding boxes around each seed for labeling it with its position and its germination state (germinated, non-germinated), as shown in Fig. 1b. One seed was classified as germinated, if there was a radicle visible, that emerged from the seed coat. We used more than 800 seeds for each of the three species (totaling 2449 seeds for all three species) to train and validate the germination classifier. This resulted in approximately 24,000 images of 2449 individual seeds, as summarized in Table 1. Between six and 12 seeds of a single species were placed on a single petri dish to capture the germination process for a maximum of 48 h after initial water contact. This resulted in a maximum of 97 longitudinal images per petri dish. However, the total number of images varied between petri dishes and species due to different technical reasons (e.g. germination was aborted due to dried out petri dishes). Additional summary statistics of the generated datasets for each of the species can be found in Additional file 1: Tables S1–S3.

**Table 1:** Summary of the annotated datasets used in this study

	Petri dishes	Seeds	Images
*Pennisetum glaucum*	82	824	7954
*Secale cereale*	81	811	7695
*Zea mays*	84	814	8148
Sum	247	2449	23,797

### Object detection and classification framework

Several neural networks have been proposed for object detection and classification, such as YOLO [16], SSD [17], R-CNN [18] and Faster R-CNN [19]. We selected Faster R-CNN in order to detect multiple seeds within an image and to classify whether a seed germinated or not. Faster R-CNN consists of two neural networks, a Region Proposal Network (RPN), to suggest Regions of Interest (ROIs) where objects (seeds) are most likely be located, and a convolutional neural network (CNN) to discriminate between germinated and non-germinated seeds. Algorithms with multiple stages, like Faster R-CNN, tend to take more time to compute, but have a higher accuracy compared to single-stage algorithms like YOLO and SSD [20]. Thus, we chose Faster-RCNN due to its higher accuracy and because real-time predictions are not necessary.

Transfer learning [15] was used to reduce the training time and to benefit from pre-computed image-based features. For this purpose, we investigated four different pre-trained CNNs, that is ResNet50, ResNet101 [21], Inception v2 [22] and Inception-ResNet v2 [23]. ResNet50/101 are two deep residual neural networks, which consists of 50 or 101 layers respectively. Residual networks use skipped connections between layers, which help in overcoming difficulties in learning, such as vanishing and exploding gradients which might lead to overfitting [21].

Inception v2 is a neural network architecture with a compact convolutional layer, where computations with different kernel sizes are done in a single layer, enabling more shallow networks. This reduces the number of network parameters and thus lowers the computational cost. Inception-ResNet v2 is a hybrid architecture that integrates parts from both inception networks and residual networks, which accelerates the training of these networks and improves the recognition performance.

### Model training and hyperparameter optimization

First we split the labeled data into a 80% training, 10% validation and 10% testing set. To prevent overfitting towards known seeds (training instances), we performed a petri dish-based stratification of the data. This stratification strategy ensures that seeds within a single petri dish are either only available during training, validation or testing (Fig. 1d). This is especially important, because the germination status of a seed might not change between certain time points (e.g. between 4-32 h), as illustrated in Fig. 1c. Second, data augmentation was used to enrich the training data by rotating, flipping and resizing the training images. This is a commonly used technique to reduce the risk of overfitting and might help to boost the performance of a classifier [24].

For each seed type we then trained four neural networks separately, each with one of the four pre-trained convolutional neural networks (ResNet50, ResNet101, Inception v2 and Inception-ResNet v2), using an internal random search for hyperparameter optimization, a dropout regularization and the Adam optimizer [25]. During the learning phase two hyperparameters have been optimized using an internal random search [26], that is the learning-rate and the dropout-rate, as summarized in Additional file 1: Table S4. The validation data of each species was used to select the model with the best performing hyperparameter pairs for each of the four neural networks. Eventually, the best performing models for each of the four networks and for each species were applied using the never used testing data to evaluate their performance and to estimate their generalization abilities. All models have been implemented using Python 3.6 and Tensorflow’s Object Detection API [20] and have been trained and tested on a Ubuntu 18.04 LTS machine with 28 Intel CPU cores, 768 GB of memory, and four GeForce RTX 2080 TI graphics cards.

### Evaluation metrics

**Fig. 2:**
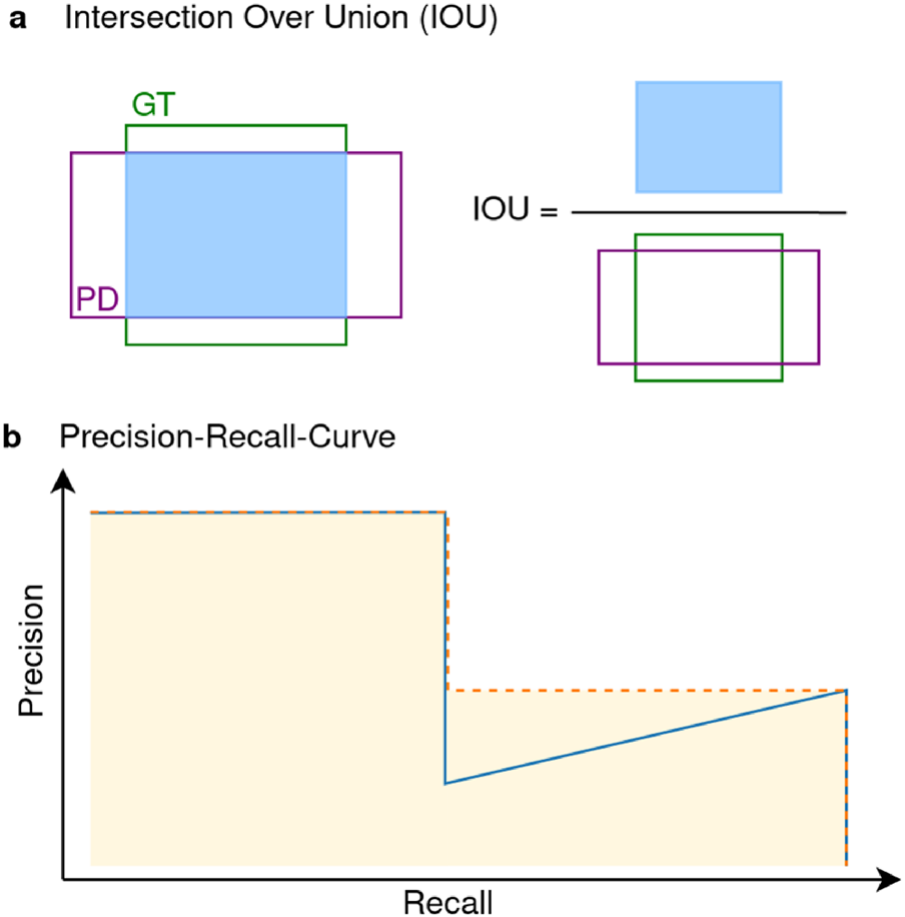
Key steps in mAP calculation for each model. **a** The IOU is the area of the overlap between the bounding box of the ground truth (GT) and the prediction (PD) divided by their union. **b** A typical Precision-Recall-Curve is shown in blue, the interpolated curve is shown in orange. The AP is the area under the curve, indicated in yellow

Different evaluation metrics have been implemented to evaluate the performance of the trained models. The mean Average Precision (mAP) is a commonly used metric for comparing the performance of computational object detection methods. The mAP can be used to compare different computational object detection methods regardless of their underlying algorithm [27]. A prediction (proposal of a region with an object) is considered as a true positive (TP), if the overlap between the bounding boxes of the prediction and the ground truth exceeds a certain threshold and if both boxes share the same label. The overlap is measured by the Intersection over Union (IOU), as illustrated in Fig. 2a. The IOU is also known as Jaccard index and is defined as:

$$IOU\left(GT,PD\right)= \frac{\left|GT\cap PD\right|}{\left|GT\cup PD\right|}=\frac{Area of the Overlap}{Area of the Union},$$

where $GT$ is the bounding box of the ground-truth, and $PD$ the bounding box of the prediction.

The precision is the ratio of true positives among all positively predicted ones, while the recall is the ratio of true positives among all positives. The Precision-Recall-Curve (PR-curve) shows the trade-off between precision and recall for different thresholds of the confidence score. The average precision (AP) is the area under an interpolated PR-Curve, as illustrated in Fig. 2b. Finally, as the AP is calculated for each class, the average of the AP values is taken for all classes to calculate the mAP.

#### Germination indices

A (cumulative) germination curve summarizes the germination process of multiple seeds (seed lot) over time (as illustrated in Fig. 6). Due to manual labor—that is mainly manual counting of germinated seeds at fixed time points—the resolution of data points might be sparse which limits germination curves-based assessments. Therefore, a number of single-value germination indices can be extracted from this curve to describe characteristics and measure the quality of a seed lot as well as to compare different seed lots [28]. We used the R package germinationmetrics [29] and focused on four indices, final germination percentage ($g$), mean germination time ($MGT$), median germination time (${t}_{50}$) and germination uncertainty ($U$), which are summarized in the following (23 additional indices have been computed and reported in the Additional file 1: Supplemental Material).

#### Final germination percentage (g)

The final germination percentage $g$ measures the number of seeds that have been germinated at the end of the experiment, e.g. after a certain time interval, that is.

$$g=\frac{{N}_{g}}{{N}_{t}}*100$$

where ${N}_{g}$ is the number of germinated seeds and ${N}_{t}$ is the total number of seeds at time $t$ after the start of the experiment [4].

#### Median germination time (t_50_)

${t}_{50}$ is the time passed until 50% of the seeds germinated and has been defined by Coolbear [30] or Farooq [31]. In this work we compute ${t}_{50}$ by Coolbear (results usings Farooq’s method can be found in the Additional file 1: Supplemental Material), that is

$${t}_{50}={T}_{i}+\frac{(\frac{N+1}{2}-{N}_{i})({T}_{j}-{T}_{i})}{{N}_{j}-{N}_{i}},$$

where $N$ is the final number of germinated seeds, ${N}_{i}$ and ${N}_{j}$ are the total number of seeds germinated in adjacent counts at time point ${T}_{i}$ and ${T}_{j}$ respectively, when ${N}_{i}<\frac{N+1}{2}<{N}_{j}$.

#### Mean germination time (MGT)

$MGT$ [32–35] estimates the weighted mean of the germination time across all observations, where the number of seeds germinated in one-time interval is used as the weight. It is defined as

$$MGT=\frac{{\sum }_{i=1}^{k}{N}_{i}{T}_{i}}{{\sum }_{i=1}^{k}{N}_{i}},$$

where ${T}_{i}$ is the time from the start of the experiment to the $i$-th interval, ${N}_{i}$ is the number of seeds germinated in the $i$-th time interval, and $k$ is the total number of time intervals.

#### Germination uncertainty (U)

The germination uncertainty $U$ estimates the synchronization of the germination across all timepoints measured [35–37] and is defined as

$$U=-{\sum }_{i=1}^{k}{f}_{i}lo{g}_{2}{f}_{i},$$

where, ${f}_{i}$ is the relative frequency of germination (estimated as ${f}_{i}=\frac{{N}_{i}}{{\sum }_{i=1}^{k}{N}_{i}}$), ${N}_{i}$ is the number of seeds germinated on the $i$-th time interval, and $k$ is the total number of time intervals. $U=0$ indicates that the germination is perfectly synchronized at each time interval (no uncertainty), while higher values of U indicate less synchronization across time points.

## Results

In this work we performed two experiments to validate the performance of the deep learning models. The aim of the first part is to evaluate the germination detection and prediction abilities of various deep learning architectures. Therefore, we used the mAP as a performance metric, which is calculated based on the whole test set for different cultivars. In the second part we estimate germination curves for each cultivar in the test set for the ground truth, the predictions and manual assessments for different time intervals. Based on the germination curves we then compare various seed germination indices, including $g, {t}_{50}, MGT$ and $U$.

### Germination detection and prediction

First, we evaluated the seed detection and germination classification abilities for three different species using Faster R-CNN and transfer learning with four different pre-trained convolutional neural network architectures (ResNet50, ResNet101, Inception v2 and Inception-ResNet v2). For each species and architecture, we selected the best performing model (measured by mAP, as summarized in “Evaluation Metrics”) on the validation set and estimated the performance on the hold-out test set. After hyperparameter optimization on the training set, Faster R-CNN with Inception-ResNet v2 was the best performing model for any species on both, the validation and the test set, as shown in Table 2. For *Zea mays*, *Secale cereale* and *Pennisetum glaucum* the models achieved a mAP (with an IOU > 50%) of 97.90%, 94.25% and 94.21% on the complete test set, respectively. Additional file 1: Supplementary Tables S5–S7 summarize the model performances for different hyperparameter combinations for each architecture and species.

**Table 2:** mAP for the validation and test sets for different model architectures

	ResNet50		ResNet101		Inception v2		Inception-ResNet v2	
	val	test	val	test	val	test	val	test
*Pennisetum glaucum*	85.80	93.66	87.62	93.66	86.16	93.06	88.91	*94.25*
*Secale cereale*	89.99	91.83	91.58	92.70	90.07	91.48	92.67	*94.21*
*Zea mays*	96.21	96.29	96.54	96.69	95.81	95.62	97.48	*97.90*

Results (in %) on the validation set are indicated by val and results on the test set are indicated by test. Italic values indicate the model with the highest mAP for each seed type

**Fig. 3:**
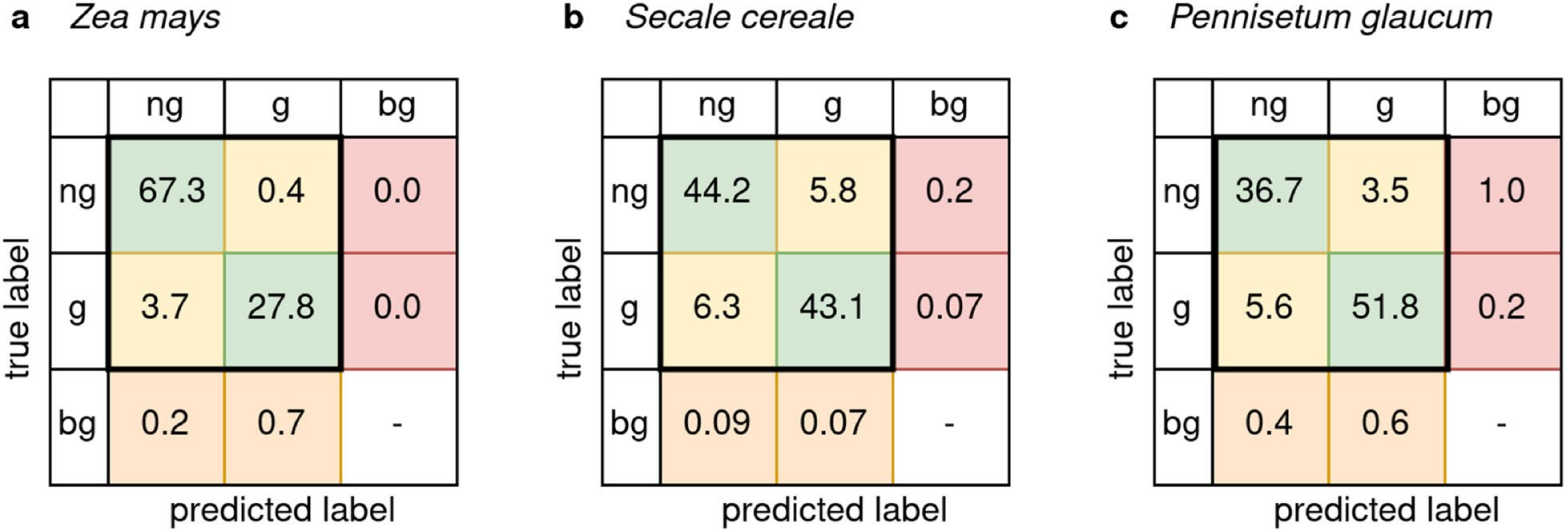
Normalized confusion matrix of test sets in percent for Inception-ResNet v2. True germination state as rows, predicted state as columns for the respective Inception-ResNet v2 model. Germinated seeds are denoted as “g”, non-germinated as “ng” and seeds which are not localized or classified by the model are denoted as “bg” (background). Green: Correct classification of the seed germination state. Yellow: Misclassification of the germination state. Orange: Incorrect localization of background as a seed (incorrect region proposal) resulting in seeds being detected multiple times. Red: Incorrect detection of a seed as background resulting in less detections than seeds present in the petri dish. **a** *Zea mays* (8809 detected instances) with a classification error of 4.1% and localization error of 0.9%. **b** *Secale cereale* (8564 detected instances) with a classification error of 12.1% and a localization error of 0.4%. **c** *Pennisetum glaucum* (8826 detected instances) with a classification error of 9.1% and a localization error of 2.2%

We computed a confusion matrix for each test set between the ground-truth and the predicted seeds. Duplicates with an $IOU>0.5$ have been removed and the confusion matrix was normalized by the number of detected instances. An additional class bg (background) is introduced to assess localization errors, as shown in Fig. 3. In addition to misclassifications (yellow), two kinds of localization errors could be observed. First, the background was wrongly localized and classified as a seed (orange), resulting in one seed being detected multiple times. Second, one seed was missed (not localized) and thus not classified (red), resulting in two neighboring seeds detected as one. A total of 2522 out of 26,010 seeds have been misclassified among all three species using the Inception-ResNet v2. Using the confusion matrix, it is possible to estimate the difficulty to detect the germination state of different seed types. Figure 3a shows the normalized confusion matrix for *Zea mays* with a classification error of 4.1% (yellow) and a localization error of 0.9% (orange + red). The model for *Secale cereale* misclassified the germination state more often (12.1%), but had a low localization error of 0.4%, as shown in Fig. 3b. *Pennisetum glaucum* was misclassified with an error rate of 9.1% and wrongly localized with a rate of 2.2% (Fig. 3c).

**Fig. 4:**
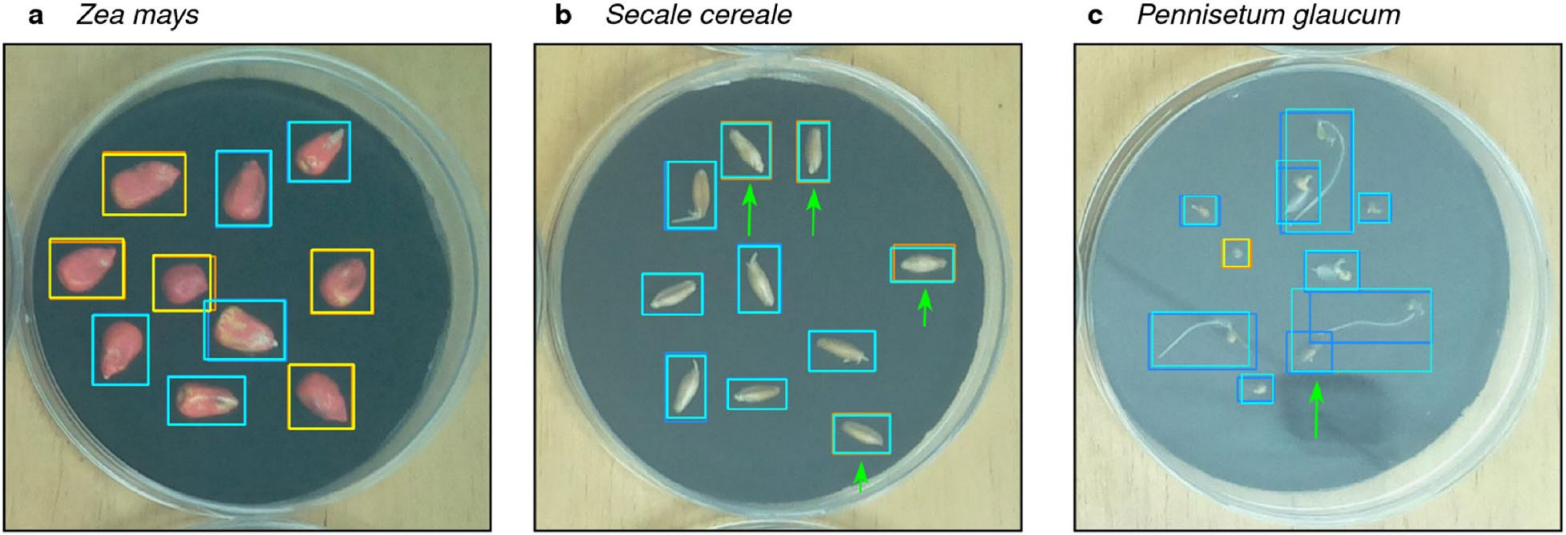
Examples of predictions on test datasets. Ground Truth is shown in dark colors (orange: non-germinated, blue: germinated) and predictions are shown in bright colors (yellow: non-germinated, cyan: germinated) **a** All seeds were correctly detected and predicted. **b** Four seeds were misclassified as germinated, as indicated by the green arrows. These errors can be rectified in the post processing step **c** Failed detection of one seed. as indicated by the green arrow. These time series were omitted when calculating germination indices

In Fig. 4a we show a positive example of correctly detected and classified seeds of *Zea mays*. Figures 4b and 3c illustrate examples when the model failed to correctly identify and predict individual seeds. In Fig. 4b four individual seeds have been misclassified as germinated (as indicated by the green arrows). In Fig. 4c one seed was not detected (localization error), as indicated by the green arrow.

### Comparison of germination indices between predicted and manual measurements

In the second experiment we estimated germination curves for each cultivar in the test set and compared different germination indices between predicted germination curves and manual assessments. Therefore, we removed outliers from the test data, which were seeds that dried out shortly after germinating and introduced an additional post-processing step to filter incorrect predictions of the best performing model.

**Fig. 5:**
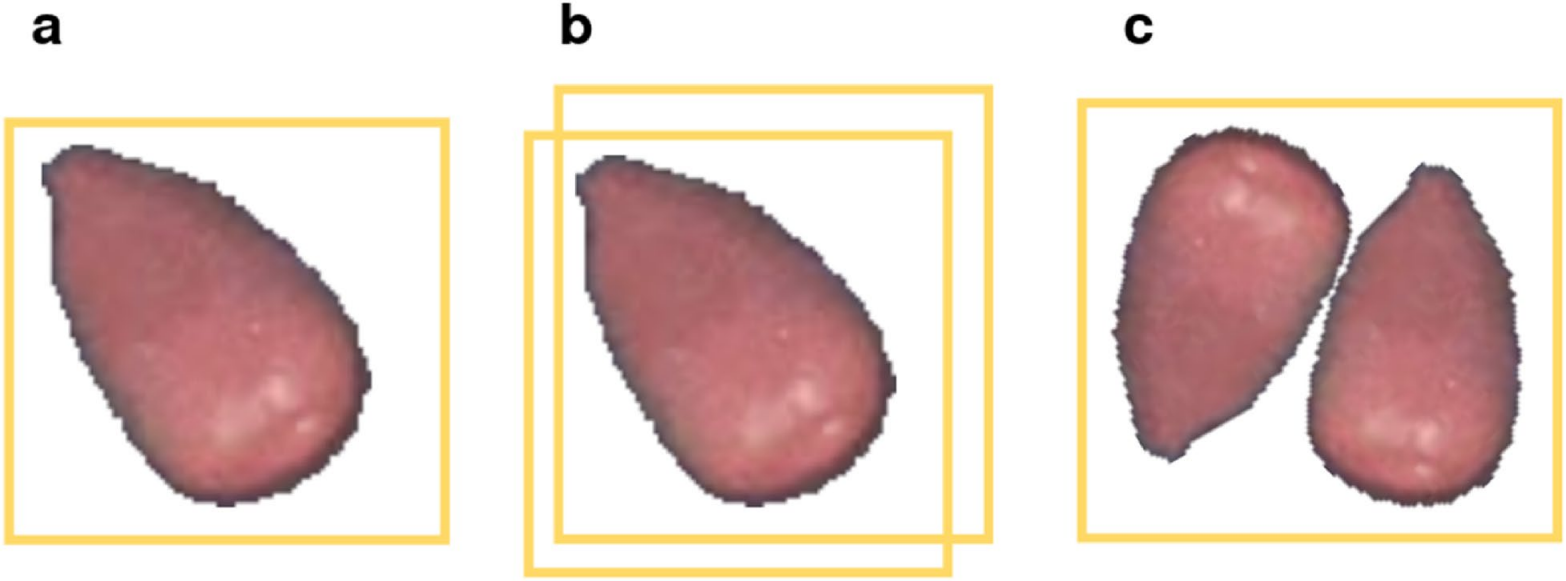
Description of different prediction errors**.** Predicted bounding box shown in yellow **a** Misclassification of a germinated seed. **b** Detection of one seed multiple times.** c** Failed detection of one seed: multiple seeds are detected as one

Different errors could appear when predicting if a seed is germinated or not, as summarized in Fig. 5. First, a simple misclassification of the germination state (Fig. 5a) occurred, mostly when the radicle was about to produce the seed coat. Misclassifications (yellow in Fig. 3) could be detected based on the time series of images of an individual seed, that is when a seed gets predicted as germinated but is classified as non-germinated in images captured shortly before and after. Second, one seed was often detected multiple times (orange in Fig. 3), which is shown with overlapping bounding boxes for one seed (Fig. 5b). These errors could be detected by calculating the IOU between all detections and removing the intersecting one. The last type of error happened rarely, if two seeds were placed too close to each other. The model predicted one bounding box for both seeds, effectively not detecting one of them (Fig. 5c). These errors (red in Fig. 3) could not reliably be detected in the post-processing step and were omitted in this experiment. In order to estimate germination curves, we first selected the best performing model for each species and classified the germination state of seeds within the first 48 h of the germination phase.

**Fig. 6:**
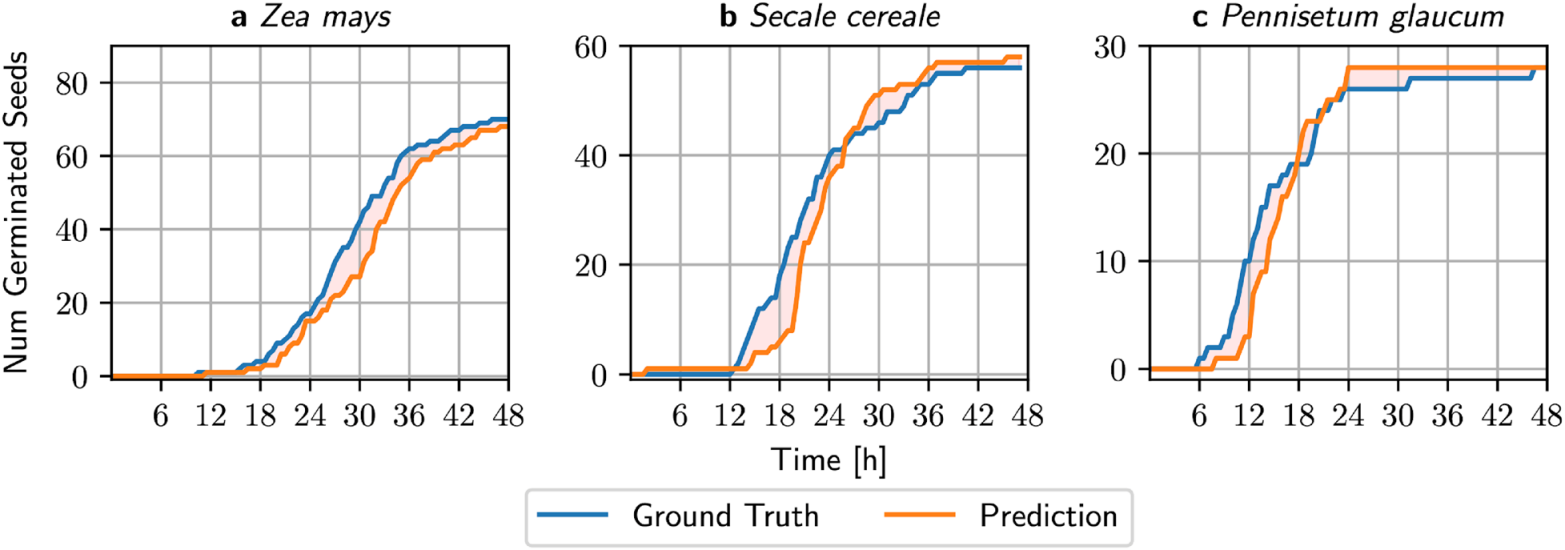
Germination curves for all three testsets. **a** *Zea Mays*, where the shape of the predicted curve resembles the ground truth curve, but is shifted because of misclassification of non-germinated seeds **b ,  c** The model does not only misclassify non-germinated, but also germinated seeds in *Secale cereale* (**b**) and *Pennisetum glaucum* (**c**)

In Fig. 6a–c we plotted the germination curves for all seeds in the test sets for both, the ground truth and the predictions for each species. The orange area indicates that the deviation between the ground-truth and predictions is rather low and that the predictions are a good approximation of the true germination curves.

These curves are used to compare the previously mentioned seed germination indices ($g, MGT, {t}_{50}, U$) between manual and predicted measurements. Predictions have then been made for every time point for which imaging data was available in the test set (every 30 min). Manual germination counts have been generated for an 6, 12 and 24 h interval. We then computed several germination indices, based on the ground truth, the predictions and the different counts from the manual assessments. The germination percentage $g$ measures the number of germinated seeds at the end of the experiments. Thus, $g$ is independent of the manual measurement rate and is the same as the ground-truth. The estimated germination percentage $g$ for *Zea mays* had a small relative error of 2.9% between the ground-truth and prediction (Additional file 1: Table S8), that is 2 out of 90 *Zea mays* seeds were misclassified as non-germinated. Similar low error rates for $g$ could be observed for the other two species, as shown in Additional file 1: Tables S9–S10.

**Fig. 7:**
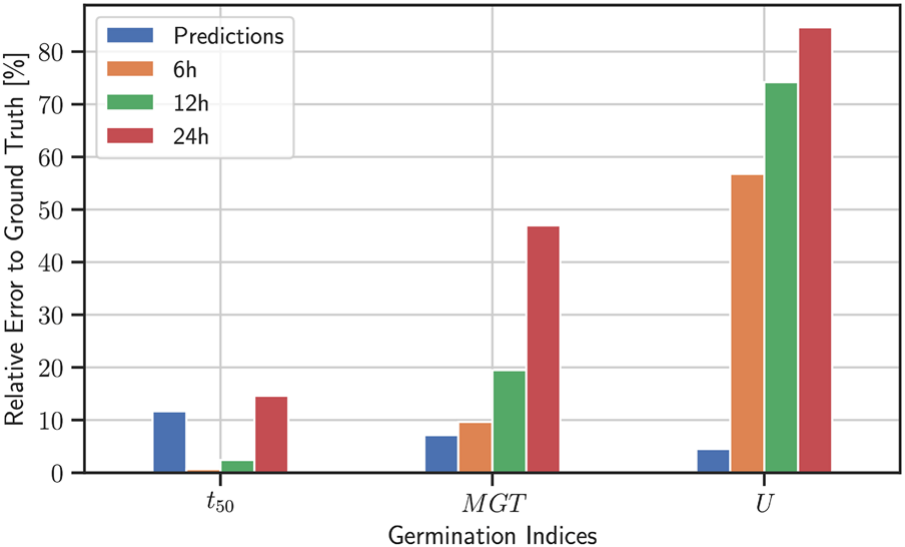
Relative error of assessment compared to the ground truth for 90 *Zea mays* seeds. Calculations based on predicted germination curves are shown in blue. Interpolations based on manual assessments are shown for 6, 12 and 24 h between each assessment and are colored in orange, green and red respectively

Figure 7 and Additional file 1: Figure S2 show the relative error between predictions and the ground-truth for *Zea mays* and the other two species respectively. For Zea mays the prediction based mean germination time ($MGT$) outperformed all manual measurements with a relative error of 7.0% compared to 9.7% error for a 6 h interval. The uncertainty $U$(based on the prediction) outperforms all manual measurements with a relative error of 3.8% compared to 55.8% error for intervals of 6 h. ${t}_{50}$(based on the predictions) outperforms the 24 h interval with a relative error of 11.3% compared to 14.5% but loses for the 6 and 12 h manual assessment with a relative error of 0.3 and 2.1% respectively. However, as mentioned above, a finer interval for the manual assessment is more time-consuming and might be unrealistic in a real-world setting. These calculations are based on absolute values for $MGT$, $U$ and ${t}_{50}$, which are summarized in Additional file 1: Table S11. Estimates of $MGT$ and $U$ using the predictions consistently outperformed estimates based on the manual counts for all three investigated seed species (see Fig. 7 and Additional file 1: Figure S2). Only ${t}_{50}$ showed better performances for the 6 h and 12 h interval compared to the predictions. However, counting germinated seeds manually every 6 h is time-consuming, cumbersome and a non-realistic scenario for most experiments. A detailed summary of a large variety of additional germination indices for each species can be also found in Additional file 1: Tables S8–S10.

## Discussion

Assessment of seed germination is an essential task for seed researchers, e.g. to measure the performance of different seed lots in order to improve the efficiency of food chains. We proposed a machine learning model based on Faster R-CNN, that automatically detects seeds within a petri dish and predicts the germination state of individual seeds. The germination process can be automatically captured by low-cost camera modules with a high frequency. Our models achieved high mAP values for all 3 datasets (> 90%), suggesting significant predictive power. Thus, our proposed method will help researchers to obtain more accurate, comparable, reproducible and less error-prone germination indices. This will enable researchers to perform various large-scale and high-throughput germination experiments with less effort, e.g. to systematically assess the effect of various abiotic and biotic factors. Further, accurately determining germination indices and other imaging-based metrics under different environments could be the basis for genome-wide association studies (GWAS). GWAS are an integral tool for studying genotype–phenotype relationships and to gain a better understanding of the genetic architecture of the underlying phenotypic variation [38,39]. These insights might help breeders to speed-up breeding cycles, which then might boost the development of plants that are e.g. more drought-resistant or produce more yield.

Conventional image analysis methods often rely on manual adjustments of color-based thresholds, especially when the experimental setup changes or different seed species have to be detected and classified. Hence, these classical methods are not well suited for large-scale germination experiments. Also, manual assessments are still utilized although being time-expensive and error-prone. A number of single-value germination indices were proposed in order to lower the frequency of assessments and to approximate germination curves. However, these approximations introduce errors that might lead to non-comparable results during the seed assessment process.

The aim of the proposed method is to automate the process of germination assessment, and minimize the manual labour. This method still needs some manual steps in terms of generating annotations for an initial training set. Nevertheless, annotating an initial set of training images might outweigh the costs of manual assessment in large scale projects, especially due to the usage of transfer learning.

Furthermore, single value germination indices can be calculated with high temporal resolution. This yields more accurate germination indices than interpolating those indices with less frequent manual observations. In addition to a higher precision, the assessment is done automatically which further reduces manual errors.

Because germination is a function over time, other machine learning approaches might be able to utilize the time at which the picture was taken, such as Convolutional LSTM (Long Short Term Memory) networks [40] or Long-term Recurrent Convolutional Networks [41], which might lead to better models and a higher mAP. High prediction accuracies have been demonstrated for similar research questions, such as seedling development detection [42]. In our work we only investigated petri dishes with uniform backgrounds for all seed types. Detecting the germination state for different greenhouse media might be more challenging and would require additional experiments. Further, a bounding box is just an approximation of the true location of an object, especially not well suitable for round shaped seeds. Using more modern methods for feature extraction, like Mask RCNN [43] (utilization of pixel-accurate locations instead of bounding boxes) could not only increase the precision, but also reduce the annotation cost. Finally, the precision of Faster R-CNN models tends to decrease for small objects [44], as indicated for the small sized seeds of *Pennisetum glaucum* (see Fig. 3). This issue could be solved by capturing the germination process with higher resolution cameras in combination with more sophisticated feature extraction methods.

Modern deep learning techniques rely on the availability of GPUs. Usually, a workstation with one or more GPUs would be sufficient to retrain our proposed method using transfer learning on other seed types. Modern single-board-systems, such as the NVIDIA Jetson Nano (similar to a Raspberry Pi, which utilizes an onboard GPU) will enable the detection and assessment of germinated seeds on-device without transferring data to a powerful computation server. This is especially useful when researchers plan to just apply pretrained models and might help to easily build up a high-throughput pipeline in greenhouses. Another alternative to physical machines could be cloud services like Amazon Web Services or Google Colab. This has the advantage that one can easily scale-up capacities if needed. The image acquisition setup in our experiments consisted of a low-cost RGB camera module and a Raspberry Pi. However, images could also be captured using more sophisticated camera setups, for example hyperspectral or NoIR cameras to investigate photoperiodism of different seeds.

## Conclusion

Our proposed method utilized modern convolutional neural network architectures to detect individual seeds with high precision and to accurately discriminate between germinated and not-germinated seeds. The models achieve a mAP of over 97% for *Zea mays* and over 94% for *Secale cereale* and *Pennisetum glaucum* on a hold-out test set. Further, single-value germination indices can be computed more accurately with the predictions of our model compared to manual assessments. Thus, our model can help to speed up the seed annotation process with lower error rates and a higher performance for larger germination experiments compared to conventional and manual methods. Further, our method can be adjusted to other seed types, petri dish media or lighting conditions by utilizing transfer learning to retrain the already pretrained models.

## Supplementary Information

**Additional file 1:** Additional tables and figures.

## Data Availability

The generated and labeled training data is freely available on Mendeley Data: http://dx.doi.org/10.17632/4wkt6thgp6.2. The code for our proposed machine learning–based model can be found on GitHub: https://github.com/grimmlab/GerminationPrediction.

## Abbreviations

CNN: Convolutional Neural Network
mAP: Mean Average Precision
ISTA: International Seed Testing Association
NBC: Naive Bayes Classifier
k-NN: K-nearest Neighbor
SVM: Support Vector Machine
ANN: Artificial Neural Network
YOLO: You Only Look Once
SSD: Single Shot Detector
R-CNN: Regions with CNN features
RPN: Region Proposal Network
TP: True Positives
IOU: Intersection Over Union
GT: Ground Truth
PD: Prediction
PR-Curve: Precision-Recall-Curve
AP: Average Precision
g: Final Germination Percentage
t_50_: Median Germination Time
MGT: Mean Germination Time
U: Germination Uncertainty

## References

[1] King T, Cole M, Farber JM, Eisenbrand G, Zabaras D, Fox EM (2017). Food safety for food security: Relationship between global megatrends and developments in food safety. Trends Food Sci Technol.

[2] Ray DK, Mueller ND, West PC, Foley JA (2013). Yield trends are insufficient to double global crop production by 2050. PLoS ONE.

[3] Marcos Filho J, Marcos FJ (2015). Seed vigor testing: an overview of the past, present and future perspective. Sci Agric Scientia Agricola.

[4] ISTA (2015). The germination test. Int Rules Seed Test.

[5] Chaugule A (2012). Application of image processing in seed technology: a survey. Int J Emerg Technol Adv Eng..

[6] Ducournau S, Feutry A, Plainchault P, Revollon P, Vigouroux B, Wagner MH (2004). An image acquisition system for automated monitoring of the germination rate of sunflower seeds. Comput Electron Agric Elsevier.

[7] Awty-Carroll D, Clifton-Brown J, Robson P (2018). Using k-NN to analyse images of diverse germination phenotypes and detect single seed germination in Miscanthus sinensis. Plant Methods.

[8] Masteling R, Voorhoeve L, Jsselmuiden IJ, Dini-Andreote F, de Boer W, Raaijmakers JM (2020). DiSCount: computer vision for automated quantification of Striga seed germination. Plant Methods.

[9] Dell’Aquila A (2009). Digital imaging information technology applied to seed germination testing. A review. Agron Sustain Dev.

[10] Joosen RV, Kodde J, Willems LA, Ligterink W, van der Plas LH, Hilhorst HW (2010). GERMINATOR: a software package for high-throughput scoring and curve fitting of Arabidopsis seed germination. Plant J.

[11] Hoffmaster AF, Xu L, Fujimura K, Bennett MA, Evans AF, McDonald MB (2005). The Ohio State University seed vigor imaging system (SVIS) for soybean and corn seedlings. Seed Technol.

[12] Škrubej U, Rozman Č, Stajnko D (2015). Assessment of germination rate of the tomato seeds using image processing and machine learning. Eur J Hortic Sci.

[13] LeCun Y, Bengio Y (1995). Convolutional networks for images, speech, and time series. Handb Brain Theory Neural Netw.

[14] Nguyen TT, Hoang VN, Le TL, Tran TH, Vu H. A vision based method for automatic evaluation of germination rate of rice seeds. In: 1st international conference on multimedia analysis and pattern recognition (MAPR). 2018. p. 1–6.

[15] Yosinski J, Clune J, Bengio Y, Lipson H, Ghahramani Z, Welling M, Cortes C, Lawrence N, Weinberger KQ (2014). How transferable are features in deep neural networks?. Advances in neural information processing systems.

[16] Redmon J, Divvala S, Girshick R, Farhadi A. You only look once: Unified, real-time object detection. In: Proceedings of the IEEE conference on computer vision and pattern recognition. 2016. p. 779–88.

[17] Liu W, Anguelov D, Erhan D, Szegedy C, Reed S, Fu C-Y, Leibe B, Matas J, Sebe N, Welling M (2016). SSD: Single Shot MultiBox Detector. Comput Vis – ECCV 2016.

[18] Girshick R, Donahue J, Darrell T, Malik J. Rich feature hierarchies for accurate object detection and semantic segmentation. In: Proceedings of the IEEE conference on computer vision and pattern recognition. 2014. p. 580–7.

[19] Ren S, He K, Girshick R, Sun J (2016). Faster r-cnn: towards real-time object detection with region proposal networks. IEEE Trans Pattern Anal Machine Intell..

[20] Huang J, Rathod V, Sun C, Zhu M, Korattikara A, Fathi A, et al. Speed/accuracy trade-offs for modern convolutional object detectors. In: Proceedings of the IEEE conference on computer vision and pattern recognition. 2017. p. 7310–11.

[21] He K, Zhang X, Ren S, Sun J. Deep residual learning for image recognition. In: Proceedings of the IEEE conference on computer vision and pattern recognition. 2016. p. 770–8.

[22] Szegedy C, Vanhoucke V, Ioffe S, Shlens J, Wojna Z. Rethinking the inception architecture for computer vision. In: Proceedings of the IEEE conference on computer vision and pattern recognition. 2016. p. 2818–26.

[23] Szegedy C, Ioffe S, Vanhoucke V, Alemi AA. Inception-v4, Inception-ResNet and the impact of residual connections on learning. In: 4th international conference on learning representations. 2016.

[24] Shorten C, Khoshgoftaar TM (2019). A survey on image data augmentation for deep learning. J Big Data.

[25] Kingma DP, Ba J. Adam: a method for stochastic optimization. In: 3rd international conference on learning representations. 2015.

[26] Bergstra J, Bengio Y (2012). Random search for hyper-parameter optimization. J Mach Learn Res.

[27] Lin T-Y, Maire M, Belongie S, Hays J, Perona P, Ramanan D, Fleet D, Pajdla T, Schiele B, Tuytelaars T (2014). Microsoft COCO: common objects in context. Comput Vis—ECCV 2014.

[28] Ranal MA, de Santana DG (2006). How and why to measure the germination process?. Rev Bras Bot.

[29] Aravind J, Vimala Devi S, Radhamani J, Jacob SR, Kalyani S. Germinationmetrics: seed germination indices and curve fitting. 2020.

[30] Coolbear P, Francis A, Grierson D (1984). The effect of low temperature pre-sowing treatment on the germination performance and membrane integrity of artificially aged tomato seeds. J Exp Bot.

[31] Farooq M, Basra SMA, Ahmad N, Hafeez K (2005). Thermal hardening: a new seed vigor enhancement tool in rice. J Integr Plant Biol.

[32] Edmond JB, Drapala WJ (1958). The effects of temperature, sand and soil, and acetone on germination of okra seed. Proc Am Soc Hortic Sci.

[33] Czabator FJ (1962). Germination value: an index combining speed and completeness of pine seed germination. For Sci.

[34] Ellis RH, Roberts EH (1980). Improved equations for the prediction of seed longevity. Ann Bot.

[35] Labouriau LG. Uma nova linha de pesquisa na fisiologia da germinação das sementes. In: Anais do XXXIV Congresso Nacional de Botânica. 1983. p. 11–50.

[36] Labouriau LG, Viladares MEB (1976). On the germination of seeds of Calotropis procera (Ait.) Ait. f. An Acad Bras Ciênc..

[37] Shannon CE (1948). A mathematical theory of communication. Bell Syst Tech J.

[38] Gumpinger AC, Roqueiro D, Grimm DG, Borgwardt KM (2018). Methods and tools in genome-wide association studies. Comput Cell Biol.

[39] Togninalli M, Seren Ü, Freudenthal JA, Monroe JG, Meng D, Nordborg M (2020). AraPheno and the AraGWAS Catalog 2020: a major database update including RNA-Seq and knockout mutation data for Arabidopsis thaliana. Nucleic Acids Res.

[40] Xingjian SHI, Chen Z, Wang H, Yeung D-Y, Wong W-K, Woo W (2015). Convolutional LSTM network: A machine learning approach for precipitation nowcasting. Adv Neural Inf Process Syst.

[41] Donahue J, Anne Hendricks L, Guadarrama S, Rohrbach M, Venugopalan S, Saenko K, et al. Long-term recurrent convolutional networks for visual recognition and description. In: Proceedings of the IEEE conference on computer vision and pattern recognition. 2015. p. 2625–34. 10.1109/TPAMI.2016.2599174 27608449

[42] Samiei S, Rasti P, Ly VuJ, Buitink J, Rousseau D (2020). Deep learning-based detection of seedling development. Plant Methods.

[43] He K, Gkioxari G, Dollar P, Girshick R. Mask r-cnn. In: Proceedings of the IEEE international conference on computer vision. 2017. p. 2961–9.

[44] Zhang L, Lin L, Liang X, He K, Leibe B, Matas J, Sebe N, Welling M (2016). Is Faster R-CNN doing well for pedestrian detection?. Computer Vision—ECCV 2016: 14th European Conference, Amsterdam, The Netherlands, October 11–14, 2016 Proceedings Part II.

