# Supplementary material for: Accurate machine learning-based germination detection, prediction and quality assessment of three grain crops
Source: Plant Methods. 2020 Dec 22;16:157. doi: 10.1186/s13007-020-00699-x (PMC7754596; doi:10.1186/s13007-020-00699-x)
Supplement: Supplementary file 1 — Additional file 1: Additional tables and figures. [file 13007_2020_699_MOESM1_ESM.pdf]

Supplementary Information

Accurate Machine Learning–Based Germination Detection, Prediction and Quality Assessment of Three Grain Crops

Nikita Genze[1,2], Richa Bharti[1,2], Michael Grieb[4], Sebastian J. Schultheiss[5], Dominik G. Grimm[1,2,3,*]

[1] Technical University of Munich, TUM Campus Straubing for Biotechnology and Sustainability, Bioinformatics, Schulgasse 22, 94315 Straubing, Germany

[2] Weihenstephan-Triesdorf University of Applied Sciences, Petersgasse 18, 94315 Straubing, Germany

[3] Technical University of Munich, Department of Informatics, Boltzmannstr. 3, 85748 Garching, Germany

[4] Technology and Support Centre in the Centre of Excellence for Renewable Resources (TFZ), Schulgasse 20, 94315 Straubing, Germany

[5] Computomics GmbH, Eisenbahnstr. 1, 72072 Tübingen, Germany

[*] Correspondence: dominik.grimm@hswt.de

Supplementary Information

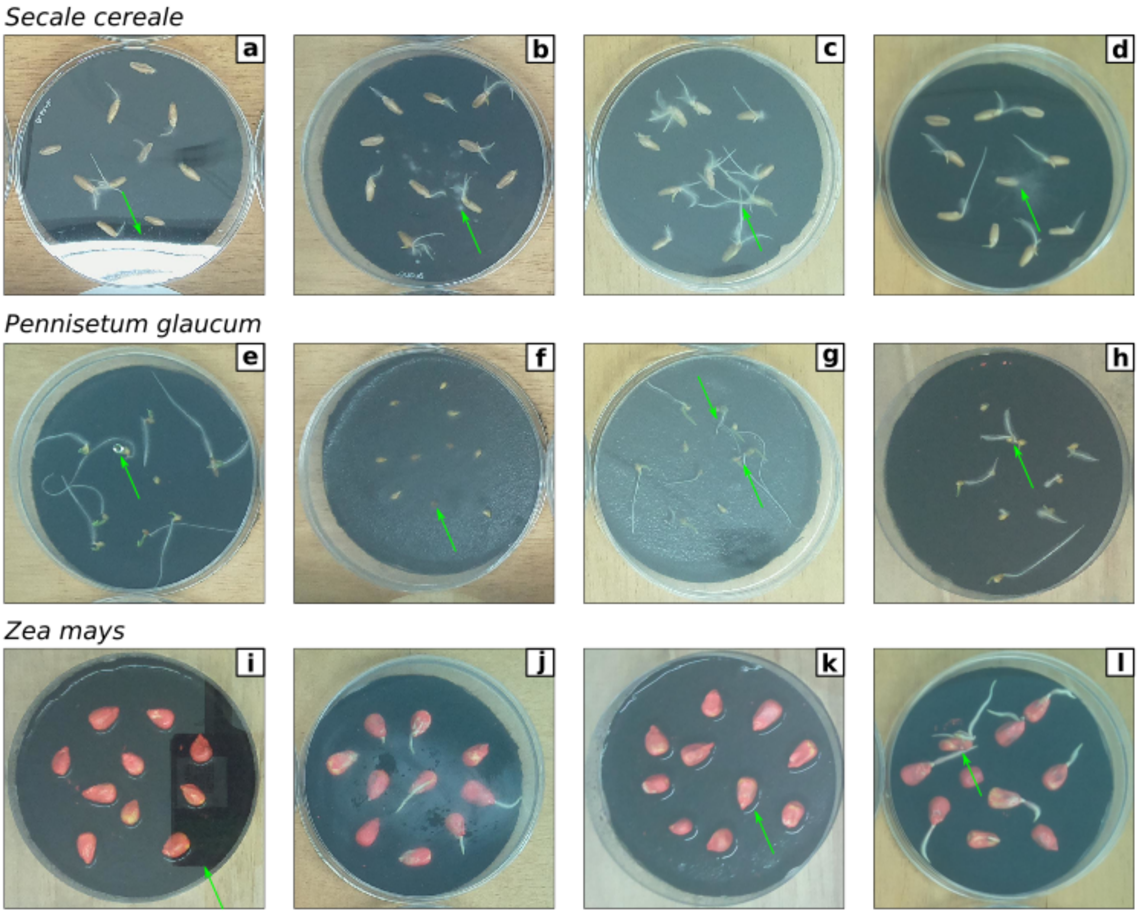

Supplementary Figure S1 Different issues while image acquisition process. Important parts are indicated by green arrows **a** Light source is reflected on the lid **b** Mold growing inside the lid **c** Radicles of multiple seeds intersecting each other **d** Irregular germination, which doesn't result in a clear radicle **e** Water drop on the lid after radicle touches it **f** Poorly visible seed due to condensation on the lid **g** Intersection of different radicles **h** Different seeds too close to each other **i** Reflection of the camera module in images **j** Condensation inside petri dishes **k** Reflections of water around seeds (at the beginning of the germination experiment) **l** Radicle of one seed touches the coat of another seed

Supplementary Table S1 Additional statistics of the dataset for *Secale cereale*.

	# time series	# images	# non-germinated	# germinated	ratio non-germinated
train	64 (640 seeds)	6080	28581	32219	0.47
eval	8 (81 seeds)	760	3800	3897	0.494
test	9 (90 seeds)	855	4303	4247	0.503
sum	81 (811 seeds)	7695	36684	40363	-

Supplementary Table S2 Additional statistics of the dataset for *Pennisetum glaucum*.

	# time series	# images	# non-germinated	# germinated	ratio non-germinated
train	65 (654 seeds)	6305	24883	38555	0.392
eval	8 (80 seeds)	776	3195	4565	0.412
test	9 (90 seeds)	873	3640	5090	0.417
sum	82 (824 seeds)	7954	31718	48210	-

Supplementary Table S3 Additional statistics of the dataset for *Zea mays*.

	# time series	# images	# non-germinated	# germinated	ratio non-germinated
train	67 (651 seeds)	6499	46216	16931	0.732
eval	8 (73 seeds)	776	4818	2263	0.68
test	9 (90 seeds)	873	5957	2773	0.682
sum	84 (814 seeds)	8148	56995	21963	-

Supplementary Table S4 Different hyperparameter pairs that were used while training. The hyperparameter set (HS) is a short reference in following results. The learning rate (lr) is used by gradient descent, the dropout-keep-probability (dkp) is the parameter used in Tensorflow's object detection api and is calculated as dkp = 1 - dropout rate.

	HS1	HS2	HS3	HS4	HS5	HS6	HS7	HS8	HS9	HS10	HS11	HS12
lr	3.6e-3	2.9e-3	1.2e-3	2.1e-4	6.5e-5	2.9e-5	5.3e-6	2.3e-6	9.9e-7	7.3e-7	5.4e-7	4.5e-7
dkp	0.51	0.687	0.529	0.801	0.866	0.652	0.646	0.716	0.916	0.591	0.578	0.806

Supplementary Table S5 Results of model evaluation for *Pennisetum glaucum*.

	ResNet50			ResNet101			Inception v2			IncResNet v2		
HS	Step [k]	loss	mAP [%]	Step [k]	loss	mAP [%]	Step [k]	loss	mAP [%]	Step [k]	loss	mAP [%]
1	2	0.7113	0	26	0.7489	0	9	0.6705	0	-	-	-
2	42	0.8094	0	-	-	-	7	0.6281	0	4	0.6389	0
3	15	0.9203	84.17	34	0.5059	0	5 (7)	0.6808	85.1 (85.52)	3	0.9338	85.11
4	4	0.6472	85.25	5	0.591	84.49	3	0.5996	84.72	**6**	**0.5513**	**88.91**
5	3	0.5832	83.86	6	0.4951	84.29	5	0.6223	85.64	47	0.7524	88.78
**6**	**7**	**0.6379**	**85.8**	**30**	**0.6068**	**87.62**	3	0.6876	85.01	14	0.6614	87.37
7	4	0.7746	83.25	4	0.8919	82.78	**11**	**0.9271**	**86.16**	14	0.7816	85.23
8	14	0.9115	83.65	5	0.8158	82.72	13	0.9606	82.82	12	0.809	86.04
9	10	0.825	83.97	12	0.9518	83.31	33	0.7759	84.77	1	0.6806	58.79
10	14	0.7812	84.22	16	0.7929	81.91	50	0.7333	84.28	1	0.5781	4
11	37	0.7531	84.25	18	0.7828	82.93	50	0.828	83.61	1	0.6939	0
12	24	1.09	83.97	37	0.8376	82.08	50	0.9884	83.42	1	0.6248	0

Supplementary Table S6 Results of model evaluation for *Secale cereale*.

	ResNet50			ResNet101			Inception v2			IncResNet v2		
HS	Step [k]	loss	mAP [%]	Step [k]	loss	mAP [%]	step	loss	mAP	Step [k]	loss	mAP [%]
1	48	0.4966	74.80	-	-	-	5	0.4649	0	-	-	-
2	39	0.7404	0	-	-	-	7	0.6691	0	-	-	-
3	9	0.6046	80.27	11	0.4369	0	9	0.2848	84.87	5	0.5216	86.78
4	**4**	**0.5124**	**89.99**	8	0.4019	90.05	5	0.4071	88.36	7	0.4793	91.61
5	6	0.3007	88.88	**9 (10)**	**0.3054**	**91.58 (90.8)**	**9**	**0.3448**	**90.07**	**21**	**0.437**	**92.67**
6	7	0.4335	88.53	9	0.4463	90.84	23	0.5838	88.15	8	0.3728	91.86
7	3	0.6217	87.43	15	0.4957	88.84	7	0.6704	86.03	17	0.5997	90.02
8	6	0.7103	86.67	13	0.5551	84.76	9	0.552	85.55	21	0.4061	88.81
9	12	0.6099	86.52	10	0.6258	84.75	31	0.6346	87.04	46	0.4337	88.34
10	13	0.5496	87.08	11	0.7298	86.01	31	0.5201	85	45	0.4642	87.92
11	32	0.514	87.02	15	0.5993	85.42	50	0.6086	85.33	46	0.4648	91.2
12	25	0.431	89.74	13	0.6718	87.79	50	0.5352	84.73	41	0.5397	87.1

Supplementary Table S7 Results of model evaluation for *Zea mays*.

	ResNet50			ResNet101			Inception v2			IncResNet v2		
HS	Step [k]	loss	mAP [%]	Step [k]	loss	mAP [%]	Step [k]	loss	mAP	Step [k]	loss	mAP [%]
1	15	0.7848	0	-	-	-	13	0.5445	0	-	-	-
2	35	0.8653	0	-	-	-	21	0.7348	0	35	0.8071	0
3	17	0.4846	93.73	15	0.4390	93.65	13	0.374	95.69	11	0.3938	96.52
4	12	0.4770	95.21	3	0.4451	95.43	47	0.3534	95.48	8	0.3987	96.86
5	8	0.3914	96.08	11	0.3077	96.47	**5**	**0.4286**	**95.81**	**9**	**0.3728**	**97.48**
6	**12**	**0.3670**	**96.21**	**13**	**0.2542**	**96.54**	45	0.5039	95.32	6	0.3514	96.79
7	7	0.4511	93.66	9	0.4542	94.02	7	0.4634	92.58	12	0.4216	95.79
8	6	0.4331	91.24	5	0.5281	92.42	11	0.4839	92.94	20	0.5844	94.69
9	11	0.3956	90.89	8	0.5036	92.37	21	0.5279	91.91	25	0.4849	94.70
10	20	0.5159	91.45	11	0.5272	90.85	33	0.4946	93.50	26	0.4883	93.13
11	24	0.5346	90.62	10	0.5160	90.34	43	0.4563	92.39	47	0.4920	93.55
12	27	0.4552	91.12	15	0.6183	91.21	47	0.4217	93.24	34	0.4865	95.15

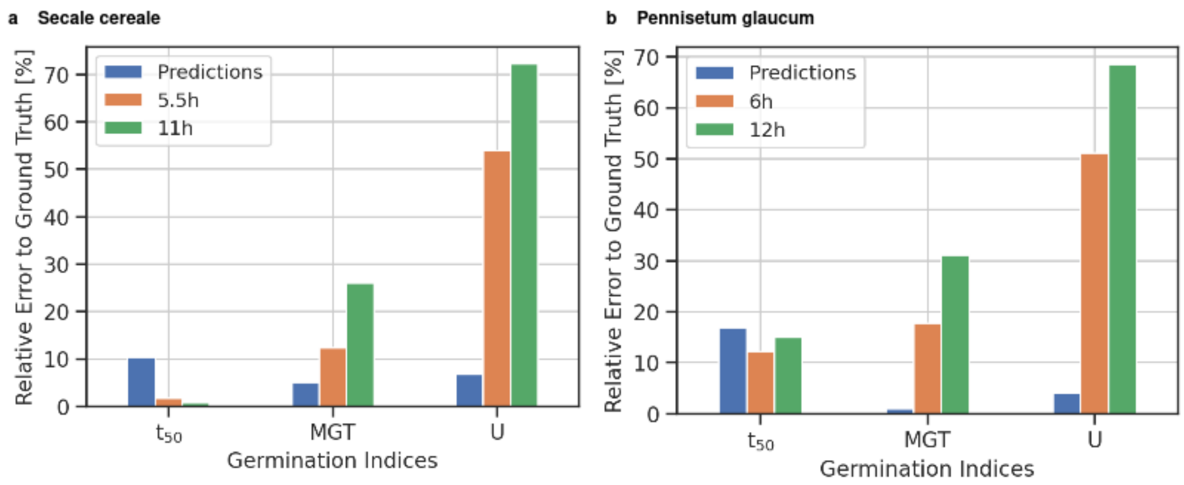

Supplementary Figure S2 Relative error compared to the ground truth. The comparison cannot be done for 22h/24h intervals, as t_{50} cannot be computed. Prediction based calculation is closer to ground truth for MGT and U **a** 60 seeds of *Secale cereale.* Intervals are measured in 5.5h and 11h intervals, as the experiment was stopped before 48h **b** 30 seeds of *Pennisetum glaucum* were compared

Supplementary Table S8 Detailed summary of germination indices for *Zea mays*. The value with the lowest error compared to the ground truth is shown in bold

	Predictions	Ground Truth	M6	M12	M24
Final Germination Percentage g [1]	76.5	77.8	**77.8**	**77.8**	**77.8**
Medium Germination Time t50 (Coolbear) [2]	3.15E+01	2.83E+01	**2.84E+01**	2.89E+01	3.24E+01
Mean Germination Time MGT [3–6]	**3.07E+01**	2.87E+01	3.15E+01	3.43E+01	4.22E+01
Germination Uncertainty U [6–8]	**4.96E+00**	5.20E+00	2.25E+00	1.34E+00	8.00E-01
Medium Germination Time t50 (Farooq) [9]	3.15E+01	2.80E+01	**2.83E+01**	2.88E+01	3.22E+01
VarGermTime [10]	5.17E+01	5.00E+01	**5.05E+01**	5.55E+01	1.07E+02
SEGermTime [10]	8.72E-01	8.45E-01	**8.50E-01**	8.90E-01	1.24E+00
CVGermTime [10,11]	2.34E-01	2.46E-01	2.26E-01	2.17E-01	**2.46E-01**
MeanGermRate [8,10]	**3.25E-02**	3.49E-02	3.18E-02	2.92E-02	2.37E-02
VarGermRate [6,10]	**5.79E-05**	7.38E-05	5.16E-05	4.01E-05	3.40E-05
SEGermRate [6,10]	**9.23E-04**	1.03E-03	8.59E-04	7.57E-04	6.97E-04
CVG [12–14]	**3.25E+00**	3.49E+00	3.18E+00	2.92E+00	2.37E+00
GermRateRecip_Coolbear [2,6,10,15]	3.17E-02	3.54E-02	**3.52E-02**	3.46E-02	3.09E-02
GermRateRecip_Farooq [6,9,10,15]	3.17E-02	3.57E-02	**3.53E-02**	3.47E-02	3.11E-02
GermSpeed_Count	**2.36E+00**	2.62E+00	**2.36E+00**	2.17E+00	1.81E+00
GermSpeedAccumulated_Count [16–19]	6.66E+01	7.86E+01	**7.19E+00**	3.97E+00	2.17E+00
GermSpeedCorrected_Normal [20]	**3.12E-02**	3.37E-02	3.04E-02	2.79E-02	2.33E-02
GermSpeedCorrected_Accumulated	**8.81E-01**	1.01E+00	9.24E-02	5.11E-02	2.79E-02
MeanGermNumber [21]	1.42E+00	1.46E+00	**1.46E+00**	**1.46E+00**	**1.46E+00**
TimsonsIndex [22]	**1.34E+03**	1.54E+03	1.75E+03	2.00E+03	2.32E+03
TimsonsIndex_Labouriau [10]	**1.78E+01**	1.98E+01	2.25E+01	2.57E+01	2.98E+01
TimsonsIndex_KhanUngar [23]	**1.40E+01**	1.60E+01	2.19E+02	5.00E+02	1.16E+03
GermRateGeorge [13,24,25]	**2.42E+03**	2.77E+03	2.63E+02	1.50E+02	8.70E+01
GermValue_Czabator [4,26]	2.75E+00	3.10E+00	**3.10E+00**	**3.10E+00**	2.63E+00
GermValue_Czabator_mod [26,27]	2.75E+00	3.10E+00	**3.10E+00**	**3.10E+00**	2.63E+00
CUGerm [14,28]	1.96E-02	2.03E-02	**2.01E-02**	1.83E-02	9.44E-03
GermSynchrony [10,29]	**2.28E-02**	1.61E-02	2.42E-01	4.71E-01	6.27E-01

Supplementary Table S9 Detailed summary of germination indices for *Secale cereale*. The value with the lowest error compared to the ground truth is shown in bold

	Predictions	Ground Truth	M5.5	M11
Final Germination Percentage g [1]	96.67	93.33	**93.33**	**93.33**
Medium Germination Time t50 (Coolbear) [2]	2.28E+01	2.06E+01	2.10E+01	**2.08E+01**
Mean Germination Time MGT [3–6]	**2.34E+01**	2.23E+01	2.50E+01	2.81E+01
Germination Uncertainty U [6–8]	4.55E+00	4.89E+00	2.25E+00	1.36E+00
Medium Germination Time t50 (Farooq) [9]	2.26E+01	2.05E+01	2.08E+01	**2.06E+01**
VarGermTime [10]	3.43E+01	5.03E+01	**5.38E+01**	6.12E+01
SEGermTime [10]	7.75E-01	9.47E-01	**9.80E-01**	1.05E+00
CVGermTime [10,11]	2.50E-01	3.18E-01	2.93E-01	2.79E-01
MeanGermRate [8,10]	4.27E-02	4.49E-02	3.99E-02	3.56E-02
VarGermRate [6,10]	1.14E-04	2.03E-04	1.37E-04	9.84E-05
SEGermRate [6,10]	1.41E-03	1.91E-03	1.56E-03	1.33E-03
CVG [12–14]	4.27E+00	4.49E+00	3.99E+00	3.56E+00
GermRateRecip_Coolbear [2,6,10,15]	4.40E-02	4.85E-02	4.76E-02	4.81E-02
GermRateRecip_Farooq [6,9,10,15]	4.42E-02	4.88E-02	4.80E-02	4.85E-02
GermSpeed_Count	2.96E+00	2.75E+00	2.42E+00	2.13E+00
GermSpeedAccumulated_Count [16–19]	7.96E+01	8.34E+01	8.03E+00	4.21E+00
GermSpeedCorrected_Normal [20]	3.11E-02	2.95E-02	2.59E-02	2.28E-02
GermSpeedCorrected_Accumulated	8.38E-01	8.94E-01	8.60E-02	4.51E-02
MeanGermNumber [21]	1.30E+00	1.27E+00	1.27E+00	1.27E+00
TimsonsIndex [22]	2.00E+03	2.07E+03	2.28E+03	2.51E+03
TimsonsIndex_Labouriau [10]	2.11E+01	2.22E+01	2.45E+01	2.69E+01
TimsonsIndex_KhanUngar [23]	2.28E+01	2.36E+01	2.85E+02	6.28E+02
GermRateGeorge [13,24,25]	2.40E+03	2.49E+03	2.49E+02	1.37E+02
GermValue_Czabator [4,26]	6.22E+00	5.92E+00	5.66E+00	5.25E+00
GermValue_Czabator_mod [26,27]	6.22E+00	5.92E+00	5.66E+00	5.25E+00
CUGerm [14,28]	2.97E-02	2.03E-02	1.89E-02	1.66E-02
GermSynchrony [10,29]	3.76E-02	2.01E-02	2.21E-01	4.24E-01

Supplementary Table S10 Detailed summary of germination indices for *Pennisetum glaucum*. The value with the lowest error compared to the ground truth is shown in bold

	Predictions	Ground Truth	6h	12h
Final Germination Percentage g [1]	**93.33**	93.33	**93.33**	**93.33**
Medium Germination Time t50 (Coolbear) [2]	1.56E+01	1.34E+01	**1.50E+01**	1.54E+01
Mean Germination Time MGT [3–6]	**1.62E+01**	1.60E+01	1.89E+01	2.10E+01
Germination Uncertainty U [6–8]	**4.07E+00**	4.24E+00	2.07E+00	1.34E+00
Medium Germination Time t50 (Farooq) [9]	1.55E+01	1.33E+01	**1.47E+01**	1.50E+01
VarGermTime [10]	1.65E+01	6.75E+01	7.12E+01	**7.07E+01**
SEGermTime [10]	7.68E-01	1.55E+00	1.60E+00	**1.59E+00**
CVGermTime [10,11]	2.51E-01	5.13E-01	**4.48E-01**	4.00E-01
MeanGermRate [8,10]	**6.18E-02**	6.24E-02	5.30E-02	4.76E-02
VarGermRate [6,10]	**2.41E-04**	1.02E-03	5.63E-04	3.63E-04
SEGermRate [6,10]	2.93E-03	6.05E-03	**4.49E-03**	3.60E-03
CVG [12–14]	**6.18E+00**	6.24E+00	5.30E+00	4.76E+00
GermRateRecip_Coolbear [2,6,10,15]	6.40E-02	7.48E-02	**6.67E-02**	6.50E-02
GermRateRecip_Farooq [6,9,10,15]	6.45E-02	7.55E-02	**6.82E-02**	6.67E-02
GermSpeed_Count	**1.85E+00**	2.11E+00	1.76E+00	1.55E+00
GermSpeedAccumulated_Count [16–19]	**6.39E+01**	6.84E+01	5.98E+00	3.25E+00
GermSpeedCorrected_Normal [20]	**1.98E-02**	2.26E-02	1.88E-02	1.66E-02
GermSpeedCorrected_Accumulated	**6.84E-01**	7.32E-01	6.41E-02	3.48E-02
MeanGermNumber [21]	**5.83E-01**	5.83E-01	**5.83E-01**	**5.83E-01**
TimsonsIndex [22]	**3.02E+03**	3.03E+03	3.28E+03	3.64E+03
TimsonsIndex_Labouriau [10]	**3.23E+01**	3.25E+01	3.51E+01	3.90E+01
TimsonsIndex_KhanUngar [23]	**3.14E+01**	3.16E+01	4.10E+02	9.10E+02
GermRateGeorge [13,24,25]	**1.81E+03**	1.82E+03	1.64E+02	9.10E+01
GermValue_Czabator [4,26]	**7.85E+00**	7.60E+00	7.02E+00	7.02E+00
GermValue_Czabator_mod [26,27]	**7.85E+00**	7.60E+00	7.02E+00	7.02E+00
CUGerm [14,28]	6.28E-02	1.54E-02	1.46E-02	**1.47E-02**
GermSynchrony [10,29]	**3.44E-02**	2.12E-02	2.46E-01	4.37E-01

Supplementary Table S11 Selection of germination indices for different timepoints in *Zea mays*. Predictions and ground-truth are based on 30 minute intervals and best value compared to ground truth is indicated in italic.

	Ground Truth	Predictions	6h (manual)	12h (manual)	24h (manual)
$MGT\ [h]$	28.7	*30.7*	31.5	34.3	42.2
$U\ [bit]$	5.2	*5.0*	2.3	1.3	0.8
$t_{50}\ [h]$	28.3	31.5	*28.4*	28.9	32.4

References

1. Association IST. International rules for seed testing. Rules 1985. Seed Sci Technol. 1985;13:299–513.
2. Coolbear P, FRANCIS A, Grierson D. The effect of low temperature pre-sowing treatment on the germination performance and membrane integrity of artificially aged tomato seeds. J Exp Bot. Oxford University Press; 1984;35:1609–1617.
3. Edmond JB, Drapala WJ. The effects of temperature, sand and soil, and acetone on germination of okra seed. Proc Am Soc Hortic Sci. 1958. p. 428–434.
4. Czabator FJ. Germination value: an index combining speed and completeness of pine seed germination. For Sci. Oxford University Press; 1962;8:386–396.
5. Ellis RH, Roberts EH. Improved equations for the prediction of seed longevity. Ann Bot. Oxford University Press; 1980;45:13–30.
6. Labouriau LG. Uma nova linha de pesquisa na fisiologia da germinação das sementes. An XXXIV Congr Nac Botânica SBB Porto Alegre. 1983. p. 11–50.
7. Shannon CE. „A mathematical theory of communication" Bell System Technical Journal, vol. 27. July Oct. 1948;
8. Labouriau LG, Viladares MEB. On the germination of seeds of Calotropis procera (Ait.) Ait. f. Anais. 1976;
9. Farooq M, Basra SMA, Ahmad N, Hafeez K. Thermal hardening: a new seed vigor enhancement tool in rice. J Integr Plant Biol. Wiley Online Library; 2005;47:187–193.
10. Ranal MA, Santana DG de. How and why to measure the germination process? Braz J Bot. SciELO Brasil; 2006;29:1–11.
11. Gomes FP. Curso de estatística experimental. Nobel; 1987.
12. Kotowski F. Temperature relations to germination of vegetable seed. 1926.
13. Nichols MA. Two approaches to the study of germination data. Proc Int Seed Test Ass. 1968;33:531–540.
14. Bewley JD, Black M. Seeds: physiology of development and germination. Springer Science & Business Media; 2013.
15. Went FW. The experimental control of plant growth. Exp Control Plant Growth. Chronica Botanica Co., Waltham, Mass., USA & The Ronald Press Co., New York; 1957;17.
16. Bradbeer JW. Seed dormancy and germination. Springer Science & Business Media; 2013.
17. Wardle DA, Ahmed M, Nicholson KS. Allelopathic influence of nodding thistle (Carduus nutans L.) seeds on germination and radicle growth of pasture plants. N Z J Agric Res. Taylor & Francis; 1991;34:185–191.
18. Haugland E, Brandsaeter LO. Experiments on bioassay sensitivity in the study of allelopathy. J Chem Ecol. Springer; 1996;22:1845–1859.
19. de Santana DG, Ranal M. Análise da germinação: um enfoque estatístico. UnB; 2004.
20. Evetts LL, Burnside OC. Germination and seedling development of common milkweed

and other species. Weed Sci. Cambridge University Press; 1972;20:371–378.
21. Khamassi K, Harbaoui K, Teixeira da Silva JA, Jeddi FB. Optimal germination temperature assessed by indices and models in Field bean (Vicia faba L. var. minor). Agric Conspec Sci. Agronomski fakultet Zagreb; 2013;78:131–136.
22. Timson J. New method of recording germination data. Nature. Springer; 1965;207:216–217.
23. Khan MA, Ungar IA. The effect of salinity and temperature on the germination of polymorphic seeds and growth of Atriplex triangularis Willd. Am J Bot. Wiley Online Library; 1984;71:481–489.
24. George DW. Influence of germination temperature on the expression of post-harvest dormancy in wheat. Crop Sci Abs. 1961;15.
25. Tucker H, Wright LN. Estimating Rapidity of Germination. Crop Sci. Crop Science Society of America; 1965;5:398–399.
26. Brown RF, Mayer DG. Representing cumulative germination. 2. The use of the Weibull function and other empirically derived curves. Ann Bot. Oxford University Press; 1988;61:127–138.
27. Djavanshir K, Pourbeik H. Germination value-a new formula. Silvae Genet. 1976;25:79–83.
28. Heydecker W. Seed ecology. Butterworths; 1973.
29. Primack RB. Longevity of individual flowers. Annu Rev Ecol Syst. JSTOR; 1985;15–37.
